# (4,7-Diphenyl-1,10-phenanthroline-κ^2^ *N*,*N*′)dimethyl­bis­(thio­cyanato-κ*N*)tin(IV)

**DOI:** 10.1107/S1600536811001735

**Published:** 2011-01-22

**Authors:** Ezzatollah Najafi, Mostafa M. Amini, Seik Weng Ng

**Affiliations:** a Department of Chemistry, General Campus, Shahid Beheshti University, Tehran 1983963113, Iran; b Department of Chemistry, University of Malaya, 50603 Kuala Lumpur, Malaysia

## Abstract

In the title compound, [Sn(CH_3_)_2_(NSC)_2_(C_24_H_16_N_2_)], a 1:1 adduct of dimethyl­tin diisothio­cyanate with 4,7-diphenyl-1,10-phenanthroline, the Sn^IV^ atom shows a slightly distorted octa­hedral SnC_2_N_4_ coordination. The methyl groups are *trans* to each other in the octa­hedron surrounding the metal atom [C—Sn—C = 176.61 (12)°].

## Related literature

For the ethanol-solvated di-*n*-butyl­tin dichloride adduct of the *N*-heterocycle, see: Hu *et al.* (1989 ▶).
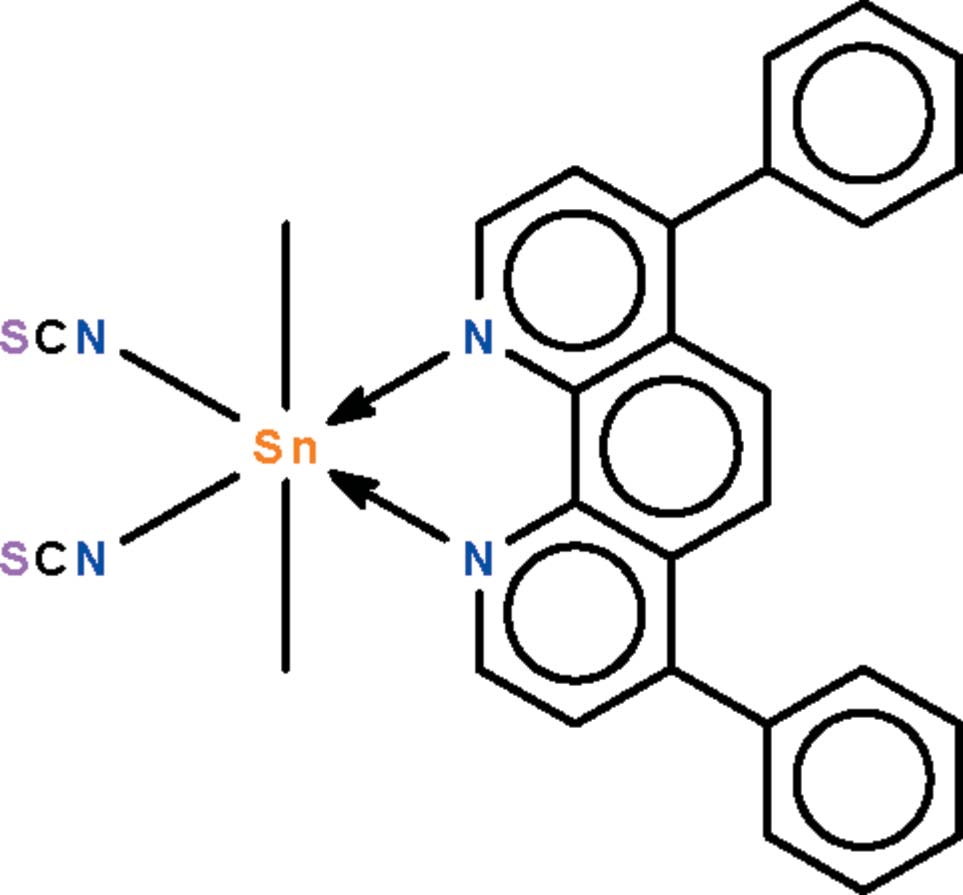


## Experimental

### 

#### Crystal data

[Sn(CH_3_)_2_(NSC)_2_(C_24_H_16_N_2_)]
*M* *_r_* = 597.31
Monoclinic, 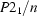

*a* = 17.1918 (2) Å
*b* = 8.1907 (2) Å
*c* = 18.3045 (3) Å
β = 98.042 (1)°
*V* = 2552.16 (8) Å^3^
*Z* = 4
Mo *K*α radiation
μ = 1.19 mm^−1^
*T* = 100 K
0.20 × 0.15 × 0.10 mm

#### Data collection

Agilent Technologies SuperNova Dual diffractometer with an Atlas detector
Absorption correction: multi-scan (*CrysAlis PRO*; Agilent Technologies, 2010 ▶) *T*_min_ = 0.797, *T*_max_ = 0.890
13167 measured reflections
5710 independent reflections
4833 reflections with *I* > 2σ(*I*)
*R*_int_ = 0.031

#### Refinement

*R*[*F* ^2^ > 2σ(*F* ^2^)] = 0.034
*wR*(*F* ^2^) = 0.084
*S* = 1.03
5710 reflections
318 parameters
H-atom parameters constrained
Δρ_max_ = 1.02 e Å^−3^
Δρ_min_ = −0.89 e Å^−3^

### 

Data collection: *CrysAlis PRO* (Agilent Technologies, 2010 ▶); cell refinement: *CrysAlis PRO*; data reduction: *CrysAlis PRO*; program(s) used to solve structure: *SHELXS97* (Sheldrick, 2008 ▶); program(s) used to refine structure: *SHELXL97* (Sheldrick, 2008 ▶); molecular graphics: *X-SEED* (Barbour, 2001 ▶); software used to prepare material for publication: *publCIF* (Westrip, 2010 ▶).

## Supplementary Material

Crystal structure: contains datablocks global, I. DOI: 10.1107/S1600536811001735/bt5462sup1.cif

Structure factors: contains datablocks I. DOI: 10.1107/S1600536811001735/bt5462Isup2.hkl

Additional supplementary materials:  crystallographic information; 3D view; checkCIF report

## Figures and Tables

**Fig. 1.**
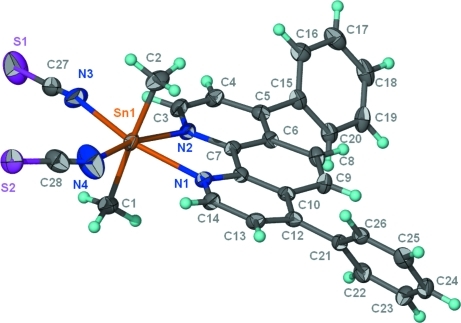
Anisotropic displacement ellipsoid plot (Barbour, 2001) of Sn(NCS)2(CH3)2(C22H16N2)at the 70% probability level; hydrogen atoms are drawn as spheres of arbitrary radius.

## supplementary crystallographic information

### Comment

Diorganotin dihalides/pseudohalides form a number of adducts with 1,10-phenanthroline and its derivatives. The dibutytlin dichloride adduct with 4,7-diphenyl-1,10-phenanthroline exists as an ethanol solvate (Hu *et al.*, 1989). The dimethyltin diisothiocyanate adduct is anhydrous (Scheme I, Fig. 1). It also features the chelated tin atom in an octahedral geometry.

### Experimental

Dimethyltin diisothiocyanate and 4,7-diphenyl-1,10-phenanthroline (1 mmol) were loaded into a convection tube. The tube was filled with dry methanol and kept at 333 K. Colorless crystals were collected from the side arm after several days.

### Refinement

H-atoms were placed in calculated positions [C—H 0.95 to 0.98 Å, *U*_iso_(H) 1.2 to 1.5*U*_eq_(C)] and were included in the refinement in the riding model approximation.

### Crystal data

**Table:**

[Sn(CH_3_)_2_(NSC)_2_(C_24_H_16_N_2_)]	*F*(000) = 1200
*M**_r_* = 597.31	*D*_x_ = 1.555 Mg m^−^^3^
Monoclinic, *P*2_1_/*n*	Mo *K*α radiation, λ = 0.71073 Å
Hall symbol: -P 2yn	Cell parameters from 6689 reflections
*a* = 17.1918 (2) Å	θ = 2.2–29.4°
*b* = 8.1907 (2) Å	µ = 1.19 mm^−^^1^
*c* = 18.3045 (3) Å	*T* = 100 K
β = 98.042 (1)°	Prism, colorless
*V* = 2552.16 (8) Å^3^	0.20 × 0.15 × 0.10 mm
*Z* = 4	

### Data collection

**Table:**

Agilent Technologies SuperNova Dual (Cu at zero) diffractometer with an Atlas detector	5710 independent reflections
Radiation source: SuperNova (Mo) X-ray Source	4833 reflections with *I* > 2σ(*I*)
Mirror	*R*_int_ = 0.031
Detector resolution: 10.4041 pixels mm^-1^	θ_max_ = 27.5°, θ_min_ = 2.3°
ω scans	*h* = −17→22
Absorption correction: multi-scan (*CrysAlis PRO*; Agilent Technologies, 2010)	*k* = −10→8
*T*_min_ = 0.797, *T*_max_ = 0.890	*l* = −23→23
13167 measured reflections	

### Refinement

**Table:**

Refinement on *F*^2^	Primary atom site location: structure-invariant direct methods
Least-squares matrix: full	Secondary atom site location: difference Fourier map
*R*[*F*^2^ > 2σ(*F*^2^)] = 0.034	Hydrogen site location: inferred from neighbouring sites
*wR*(*F*^2^) = 0.084	H-atom parameters constrained
*S* = 1.03	*w* = 1/[σ^2^(*F*_o_^2^) + (0.0372*P*)^2^ + 1.3448*P*] where *P* = (*F*_o_^2^ + 2*F*_c_^2^)/3
5710 reflections	(Δ/σ)_max_ = 0.001
318 parameters	Δρ_max_ = 1.02 e Å^−^^3^
0 restraints	Δρ_min_ = −0.89 e Å^−^^3^

### Fractional atomic coordinates and isotropic or equivalent isotropic displacement parameters (Å^2^)

**Table:**

	*x*	*y*	*z*	*U*_iso_*/*U*_eq_	
Sn1	0.430971 (9)	0.35713 (2)	0.153313 (10)	0.02007 (7)	
S1	0.39425 (6)	0.14565 (14)	−0.10042 (6)	0.0523 (3)	
S2	0.15135 (4)	0.31635 (11)	0.17421 (5)	0.03287 (19)	
N1	0.50393 (11)	0.4293 (3)	0.26740 (12)	0.0188 (5)	
N2	0.56572 (12)	0.3760 (3)	0.14104 (13)	0.0183 (5)	
N3	0.39761 (16)	0.3004 (3)	0.03302 (14)	0.0346 (6)	
N4	0.31366 (17)	0.3499 (4)	0.1919 (2)	0.0528 (9)	
C1	0.44834 (17)	0.1079 (4)	0.17985 (18)	0.0308 (7)	
H1A	0.4090	0.0722	0.2104	0.046*	
H1B	0.5011	0.0924	0.2072	0.046*	
H1C	0.4430	0.0433	0.1344	0.046*	
C2	0.41642 (17)	0.6092 (4)	0.13329 (18)	0.0291 (7)	
H2A	0.3711	0.6271	0.0953	0.044*	
H2B	0.4638	0.6534	0.1163	0.044*	
H2C	0.4076	0.6645	0.1789	0.044*	
C3	0.59521 (15)	0.3505 (4)	0.07902 (16)	0.0225 (6)	
H3	0.5613	0.3103	0.0375	0.027*	
C4	0.67354 (15)	0.3796 (3)	0.07169 (15)	0.0207 (6)	
H4	0.6919	0.3584	0.0260	0.025*	
C5	0.72464 (14)	0.4391 (3)	0.13051 (15)	0.0178 (5)	
C6	0.69505 (14)	0.4625 (3)	0.19856 (15)	0.0169 (5)	
C7	0.61521 (14)	0.4290 (3)	0.20143 (14)	0.0174 (5)	
C8	0.74140 (14)	0.5247 (3)	0.26383 (15)	0.0196 (6)	
H8	0.7944	0.5553	0.2619	0.023*	
C9	0.71173 (14)	0.5409 (4)	0.32806 (15)	0.0202 (6)	
H9	0.7451	0.5773	0.3707	0.024*	
C10	0.63136 (14)	0.5044 (3)	0.33323 (15)	0.0171 (5)	
C11	0.58269 (13)	0.4536 (3)	0.26895 (14)	0.0165 (5)	
C12	0.59669 (14)	0.5299 (3)	0.39857 (15)	0.0176 (5)	
C13	0.51612 (14)	0.5121 (4)	0.39372 (15)	0.0197 (6)	
H13	0.4906	0.5346	0.4354	0.024*	
C14	0.47207 (14)	0.4613 (4)	0.32769 (15)	0.0208 (6)	
H14	0.4169	0.4491	0.3259	0.025*	
C15	0.80687 (14)	0.4777 (3)	0.11987 (15)	0.0184 (6)	
C16	0.81958 (15)	0.5699 (4)	0.05879 (16)	0.0230 (6)	
H16	0.7761	0.6070	0.0251	0.028*	
C17	0.89580 (17)	0.6081 (4)	0.04668 (18)	0.0286 (7)	
H17	0.9042	0.6746	0.0060	0.034*	
C18	0.95916 (16)	0.5487 (4)	0.09433 (17)	0.0290 (7)	
H18	1.0111	0.5734	0.0858	0.035*	
C19	0.94723 (15)	0.4538 (4)	0.15416 (16)	0.0249 (6)	
H19	0.9910	0.4115	0.1860	0.030*	
C20	0.87128 (14)	0.4198 (4)	0.16795 (15)	0.0204 (6)	
H20	0.8633	0.3574	0.2100	0.024*	
C21	0.64392 (14)	0.5755 (3)	0.46954 (14)	0.0173 (5)	
C22	0.71205 (15)	0.4890 (4)	0.49706 (16)	0.0228 (6)	
H22	0.7295	0.4020	0.4691	0.027*	
C23	0.75401 (15)	0.5293 (4)	0.56471 (16)	0.0252 (6)	
H23	0.7995	0.4684	0.5834	0.030*	
C24	0.73016 (16)	0.6579 (4)	0.60538 (16)	0.0246 (6)	
H24	0.7591	0.6851	0.6518	0.030*	
C25	0.66399 (15)	0.7464 (4)	0.57809 (15)	0.0238 (6)	
H25	0.6484	0.8365	0.6053	0.029*	
C26	0.62034 (14)	0.7044 (3)	0.51140 (15)	0.0185 (6)	
H26	0.5740	0.7637	0.4939	0.022*	
C27	0.39747 (15)	0.2339 (4)	−0.02074 (18)	0.0276 (7)	
C28	0.24557 (18)	0.3359 (4)	0.18306 (19)	0.0323 (7)	

### Atomic displacement parameters (Å^2^)

**Table:**

	*U*^11^	*U*^22^	*U*^33^	*U*^12^	*U*^13^	*U*^23^
Sn1	0.01425 (10)	0.02700 (13)	0.01784 (12)	−0.00130 (7)	−0.00165 (7)	0.00103 (8)
S1	0.0643 (6)	0.0581 (7)	0.0384 (5)	−0.0235 (5)	0.0209 (5)	−0.0208 (5)
S2	0.0185 (3)	0.0407 (5)	0.0401 (5)	−0.0003 (3)	0.0063 (3)	0.0046 (4)
N1	0.0156 (10)	0.0238 (13)	0.0168 (11)	−0.0006 (9)	0.0011 (9)	0.0047 (10)
N2	0.0172 (10)	0.0224 (13)	0.0150 (11)	−0.0005 (9)	0.0010 (9)	0.0011 (10)
N3	0.0547 (17)	0.0268 (15)	0.0194 (14)	0.0013 (13)	−0.0052 (12)	−0.0013 (12)
N4	0.0261 (15)	0.053 (2)	0.083 (3)	−0.0066 (13)	0.0204 (16)	−0.0049 (18)
C1	0.0262 (14)	0.0320 (18)	0.0321 (17)	−0.0039 (12)	−0.0038 (13)	0.0119 (14)
C2	0.0287 (15)	0.0300 (18)	0.0275 (16)	0.0092 (12)	0.0004 (13)	0.0032 (14)
C3	0.0210 (13)	0.0283 (17)	0.0169 (14)	−0.0005 (11)	−0.0017 (11)	0.0015 (12)
C4	0.0206 (13)	0.0257 (16)	0.0161 (14)	0.0016 (11)	0.0037 (11)	0.0004 (12)
C5	0.0189 (12)	0.0152 (14)	0.0198 (14)	0.0023 (10)	0.0042 (10)	0.0011 (11)
C6	0.0151 (11)	0.0163 (14)	0.0196 (13)	0.0001 (10)	0.0038 (10)	−0.0008 (11)
C7	0.0159 (12)	0.0173 (14)	0.0184 (13)	0.0011 (10)	0.0000 (10)	0.0032 (11)
C8	0.0150 (11)	0.0192 (14)	0.0248 (15)	−0.0007 (10)	0.0042 (10)	−0.0043 (12)
C9	0.0151 (12)	0.0243 (15)	0.0207 (14)	−0.0010 (10)	0.0008 (10)	−0.0038 (12)
C10	0.0184 (12)	0.0133 (13)	0.0194 (14)	0.0013 (10)	0.0026 (10)	0.0025 (11)
C11	0.0151 (11)	0.0155 (14)	0.0185 (13)	−0.0004 (10)	0.0013 (10)	0.0016 (11)
C12	0.0175 (12)	0.0164 (14)	0.0191 (14)	0.0017 (10)	0.0027 (10)	0.0028 (11)
C13	0.0189 (12)	0.0241 (15)	0.0170 (14)	0.0007 (11)	0.0054 (10)	0.0031 (12)
C14	0.0152 (12)	0.0295 (16)	0.0177 (14)	−0.0023 (11)	0.0025 (10)	0.0041 (12)
C15	0.0190 (12)	0.0161 (14)	0.0212 (14)	0.0004 (10)	0.0070 (11)	−0.0067 (11)
C16	0.0250 (13)	0.0191 (15)	0.0254 (15)	0.0019 (11)	0.0057 (11)	−0.0014 (13)
C17	0.0326 (15)	0.0247 (17)	0.0315 (17)	−0.0055 (12)	0.0148 (14)	−0.0041 (14)
C18	0.0230 (14)	0.0298 (18)	0.0365 (18)	−0.0092 (12)	0.0125 (13)	−0.0131 (15)
C19	0.0166 (12)	0.0310 (17)	0.0266 (16)	−0.0002 (11)	0.0021 (11)	−0.0131 (13)
C20	0.0213 (12)	0.0199 (15)	0.0203 (14)	−0.0014 (11)	0.0047 (11)	−0.0064 (12)
C21	0.0174 (12)	0.0188 (14)	0.0160 (13)	−0.0029 (10)	0.0031 (10)	0.0004 (12)
C22	0.0211 (13)	0.0184 (15)	0.0281 (16)	−0.0003 (10)	0.0014 (11)	−0.0013 (12)
C23	0.0179 (12)	0.0290 (17)	0.0269 (16)	0.0004 (11)	−0.0030 (11)	0.0037 (13)
C24	0.0205 (13)	0.0340 (18)	0.0191 (15)	−0.0082 (12)	0.0016 (11)	−0.0003 (13)
C25	0.0271 (14)	0.0257 (16)	0.0204 (15)	−0.0036 (12)	0.0102 (11)	−0.0046 (13)
C26	0.0167 (12)	0.0197 (14)	0.0192 (14)	−0.0005 (10)	0.0034 (10)	0.0016 (12)
C27	0.0185 (13)	0.0306 (18)	0.0321 (18)	−0.0043 (12)	−0.0017 (12)	0.0093 (15)
C28	0.0285 (16)	0.0304 (18)	0.040 (2)	−0.0006 (12)	0.0132 (14)	0.0021 (15)

### Geometric parameters (Å, °)

**Table:**

Sn1—C2	2.106 (3)	C9—C10	1.430 (3)
Sn1—C1	2.110 (3)	C9—H9	0.9500
Sn1—N4	2.229 (3)	C10—C11	1.408 (4)
Sn1—N3	2.245 (3)	C10—C12	1.424 (4)
Sn1—N1	2.356 (2)	C12—C13	1.383 (3)
Sn1—N2	2.364 (2)	C12—C21	1.480 (4)
S1—C27	1.622 (3)	C13—C14	1.397 (4)
S2—C28	1.613 (3)	C13—H13	0.9500
N1—C14	1.325 (3)	C14—H14	0.9500
N1—C11	1.365 (3)	C15—C16	1.392 (4)
N2—C3	1.323 (4)	C15—C20	1.397 (4)
N2—C7	1.368 (3)	C16—C17	1.395 (4)
N3—C27	1.125 (4)	C16—H16	0.9500
N4—C28	1.165 (4)	C17—C18	1.385 (4)
C1—H1A	0.9800	C17—H17	0.9500
C1—H1B	0.9800	C18—C19	1.382 (4)
C1—H1C	0.9800	C18—H18	0.9500
C2—H2A	0.9800	C19—C20	1.392 (3)
C2—H2B	0.9800	C19—H19	0.9500
C2—H2C	0.9800	C20—H20	0.9500
C3—C4	1.392 (4)	C21—C26	1.397 (4)
C3—H3	0.9500	C21—C22	1.401 (4)
C4—C5	1.380 (4)	C22—C23	1.383 (4)
C4—H4	0.9500	C22—H22	0.9500
C5—C6	1.423 (4)	C23—C24	1.384 (4)
C5—C15	1.488 (3)	C23—H23	0.9500
C6—C7	1.408 (3)	C24—C25	1.382 (4)
C6—C8	1.434 (4)	C24—H24	0.9500
C7—C11	1.440 (4)	C25—C26	1.384 (4)
C8—C9	1.352 (4)	C25—H25	0.9500
C8—H8	0.9500	C26—H26	0.9500
			
C2—Sn1—C1	176.61 (12)	C10—C9—H9	119.2
C2—Sn1—N4	89.45 (12)	C11—C10—C12	118.4 (2)
C1—Sn1—N4	90.38 (12)	C11—C10—C9	118.2 (2)
C2—Sn1—N3	91.41 (12)	C12—C10—C9	123.1 (2)
C1—Sn1—N3	91.95 (12)	N1—C11—C10	122.2 (2)
N4—Sn1—N3	100.76 (12)	N1—C11—C7	117.7 (2)
C2—Sn1—N1	86.78 (11)	C10—C11—C7	120.1 (2)
C1—Sn1—N1	89.89 (11)	C13—C12—C10	117.6 (2)
N4—Sn1—N1	96.84 (11)	C13—C12—C21	120.3 (2)
N3—Sn1—N1	162.29 (9)	C10—C12—C21	122.1 (2)
C2—Sn1—N2	90.76 (10)	C12—C13—C14	120.2 (2)
C1—Sn1—N2	88.64 (10)	C12—C13—H13	119.9
N4—Sn1—N2	166.95 (11)	C14—C13—H13	119.9
N3—Sn1—N2	92.27 (9)	N1—C14—C13	122.9 (2)
N1—Sn1—N2	70.15 (7)	N1—C14—H14	118.6
C14—N1—C11	118.6 (2)	C13—C14—H14	118.6
C14—N1—Sn1	123.85 (16)	C16—C15—C20	119.4 (2)
C11—N1—Sn1	117.30 (16)	C16—C15—C5	118.7 (2)
C3—N2—C7	118.3 (2)	C20—C15—C5	122.0 (2)
C3—N2—Sn1	124.71 (18)	C15—C16—C17	120.4 (3)
C7—N2—Sn1	116.81 (16)	C15—C16—H16	119.8
C27—N3—Sn1	158.1 (3)	C17—C16—H16	119.8
C28—N4—Sn1	153.5 (3)	C18—C17—C16	119.6 (3)
Sn1—C1—H1A	109.5	C18—C17—H17	120.2
Sn1—C1—H1B	109.5	C16—C17—H17	120.2
H1A—C1—H1B	109.5	C19—C18—C17	120.4 (2)
Sn1—C1—H1C	109.5	C19—C18—H18	119.8
H1A—C1—H1C	109.5	C17—C18—H18	119.8
H1B—C1—H1C	109.5	C18—C19—C20	120.2 (3)
Sn1—C2—H2A	109.5	C18—C19—H19	119.9
Sn1—C2—H2B	109.5	C20—C19—H19	119.9
H2A—C2—H2B	109.5	C19—C20—C15	119.9 (3)
Sn1—C2—H2C	109.5	C19—C20—H20	120.0
H2A—C2—H2C	109.5	C15—C20—H20	120.0
H2B—C2—H2C	109.5	C26—C21—C22	118.5 (2)
N2—C3—C4	123.2 (3)	C26—C21—C12	120.4 (2)
N2—C3—H3	118.4	C22—C21—C12	121.1 (3)
C4—C3—H3	118.4	C23—C22—C21	120.4 (3)
C5—C4—C3	120.3 (3)	C23—C22—H22	119.8
C5—C4—H4	119.9	C21—C22—H22	119.8
C3—C4—H4	119.9	C22—C23—C24	120.4 (3)
C4—C5—C6	117.6 (2)	C22—C23—H23	119.8
C4—C5—C15	119.1 (2)	C24—C23—H23	119.8
C6—C5—C15	123.3 (2)	C25—C24—C23	119.7 (3)
C7—C6—C5	118.4 (2)	C25—C24—H24	120.2
C7—C6—C8	118.0 (2)	C23—C24—H24	120.2
C5—C6—C8	123.5 (2)	C24—C25—C26	120.4 (3)
N2—C7—C6	122.1 (2)	C24—C25—H25	119.8
N2—C7—C11	117.8 (2)	C26—C25—H25	119.8
C6—C7—C11	120.1 (2)	C25—C26—C21	120.6 (2)
C9—C8—C6	121.8 (2)	C25—C26—H26	119.7
C9—C8—H8	119.1	C21—C26—H26	119.7
C6—C8—H8	119.1	N3—C27—S1	176.9 (3)
C8—C9—C10	121.5 (2)	N4—C28—S2	177.8 (4)
C8—C9—H9	119.2		
			
C2—Sn1—N1—C14	85.1 (2)	C8—C9—C10—C11	−1.2 (4)
C1—Sn1—N1—C14	−94.3 (2)	C8—C9—C10—C12	−175.8 (3)
N4—Sn1—N1—C14	−3.9 (2)	C14—N1—C11—C10	2.8 (4)
N3—Sn1—N1—C14	169.6 (3)	Sn1—N1—C11—C10	177.00 (19)
N2—Sn1—N1—C14	177.1 (2)	C14—N1—C11—C7	−175.8 (2)
C2—Sn1—N1—C11	−88.7 (2)	Sn1—N1—C11—C7	−1.6 (3)
C1—Sn1—N1—C11	91.9 (2)	C12—C10—C11—N1	0.4 (4)
N4—Sn1—N1—C11	−177.7 (2)	C9—C10—C11—N1	−174.5 (3)
N3—Sn1—N1—C11	−4.2 (4)	C12—C10—C11—C7	179.0 (2)
N2—Sn1—N1—C11	3.29 (19)	C9—C10—C11—C7	4.1 (4)
C2—Sn1—N2—C3	−93.4 (2)	N2—C7—C11—N1	−2.8 (4)
C1—Sn1—N2—C3	89.9 (2)	C6—C7—C11—N1	175.9 (3)
N4—Sn1—N2—C3	175.8 (4)	N2—C7—C11—C10	178.6 (2)
N3—Sn1—N2—C3	−2.0 (2)	C6—C7—C11—C10	−2.8 (4)
N1—Sn1—N2—C3	−179.7 (2)	C11—C10—C12—C13	−3.6 (4)
C2—Sn1—N2—C7	81.6 (2)	C9—C10—C12—C13	171.0 (3)
C1—Sn1—N2—C7	−95.1 (2)	C11—C10—C12—C21	177.0 (3)
N4—Sn1—N2—C7	−9.3 (6)	C9—C10—C12—C21	−8.4 (4)
N3—Sn1—N2—C7	173.0 (2)	C10—C12—C13—C14	3.7 (4)
N1—Sn1—N2—C7	−4.72 (18)	C21—C12—C13—C14	−176.8 (3)
C2—Sn1—N3—C27	147.3 (7)	C11—N1—C14—C13	−2.8 (4)
C1—Sn1—N3—C27	−32.2 (7)	Sn1—N1—C14—C13	−176.6 (2)
N4—Sn1—N3—C27	−123.0 (7)	C12—C13—C14—N1	−0.5 (4)
N1—Sn1—N3—C27	63.5 (8)	C4—C5—C15—C16	47.9 (4)
N2—Sn1—N3—C27	56.5 (7)	C6—C5—C15—C16	−131.2 (3)
C2—Sn1—N4—C28	88.5 (7)	C4—C5—C15—C20	−130.0 (3)
C1—Sn1—N4—C28	−94.8 (7)	C6—C5—C15—C20	50.9 (4)
N3—Sn1—N4—C28	−2.8 (7)	C20—C15—C16—C17	−1.8 (4)
N1—Sn1—N4—C28	175.2 (7)	C5—C15—C16—C17	−179.7 (3)
N2—Sn1—N4—C28	179.5 (5)	C15—C16—C17—C18	2.4 (4)
C7—N2—C3—C4	−2.0 (4)	C16—C17—C18—C19	−0.8 (5)
Sn1—N2—C3—C4	172.9 (2)	C17—C18—C19—C20	−1.4 (4)
N2—C3—C4—C5	−0.5 (4)	C18—C19—C20—C15	2.0 (4)
C3—C4—C5—C6	2.5 (4)	C16—C15—C20—C19	−0.4 (4)
C3—C4—C5—C15	−176.6 (3)	C5—C15—C20—C19	177.5 (3)
C4—C5—C6—C7	−2.0 (4)	C13—C12—C21—C26	−47.3 (4)
C15—C5—C6—C7	177.1 (3)	C10—C12—C21—C26	132.1 (3)
C4—C5—C6—C8	−179.4 (3)	C13—C12—C21—C22	131.4 (3)
C15—C5—C6—C8	−0.3 (4)	C10—C12—C21—C22	−49.3 (4)
C3—N2—C7—C6	2.4 (4)	C26—C21—C22—C23	1.0 (4)
Sn1—N2—C7—C6	−172.9 (2)	C12—C21—C22—C23	−177.7 (3)
C3—N2—C7—C11	−178.9 (2)	C21—C22—C23—C24	−1.3 (4)
Sn1—N2—C7—C11	5.8 (3)	C22—C23—C24—C25	0.0 (4)
C5—C6—C7—N2	−0.4 (4)	C23—C24—C25—C26	1.8 (4)
C8—C6—C7—N2	177.1 (2)	C24—C25—C26—C21	−2.1 (4)
C5—C6—C7—C11	−179.0 (2)	C22—C21—C26—C25	0.8 (4)
C8—C6—C7—C11	−1.4 (4)	C12—C21—C26—C25	179.4 (2)
C7—C6—C8—C9	4.5 (4)	Sn1—N3—C27—S1	−174 (5)
C5—C6—C8—C9	−178.1 (3)	Sn1—N4—C28—S2	171 (8)
C6—C8—C9—C10	−3.2 (4)		
